# Supplementary material for: Diversity and Abundance of Potential Vectors of Rift Valley Fever Virus in the North Region of Cameroon
Source: Insects. 2020 Nov 19;11(11):814. doi: 10.3390/insects11110814 (PMC7699143; doi:10.3390/insects11110814)
Supplement: Supplementary file 1 [file insects-11-00814-s001.pdf]

S1 Table: Summary of mosquito species captured: Diversity, distribution and abundance per locality.

Mosquito genus	Mosquito species	Localities (trap nights)								Total number of mosquitoes per species	Mean M/T/N
		Bokle (10)		Garoua (13)		Lagdo (14)		Pitoa (15)			
		No.	M/T/N	No.	M/T/N	No.	M/T/N	No.	M/T/N		
Aedes	*Ae aegypti*	12	0.6	51	1.96	3	0.11	54	1.80	120	1.12
	Ae albopictus	4	0.2	22	0.85	3	0.11	3	0.10	32	0.31
	Ae circumluteolus	0	0	19	0.73	14	0.50	0	0.00	33	0.31
	Ae dalzieli	4	0.2	25	0.96	19	0.68	10	0.33	58	0.54
	Ae fowleri	5	0.25	22	0.85	36	1.29	31	1.03	94	0.85
	Ae mcintoshi	42	2.1	0	0	0	0.00		0.00	42	0.53
	Ae mucidus	0	0	0	0	5	0.18	15	0.50	20	0.17
	Ae ochraceus	6	0.3	7	0.27	32	1.14	18	0.60	63	0.58
	Ae vittatus	35	1.75	33	1.27	4	0.14	8	0.27	80	0.86
	TOTAL *Aedes*	108	0.6	179	0.76	116	0.46	139	0.51	542	0.59
Anopheles	*An. wellcomei*	0	0	0	0	0	0.00	3	0.10	3	0.03
	An coustani	0	0	0	0	27	0.96	3	0.10	30	0.27
	An funestus	12	0.6	6	0.23	13	0.46	2	0.07	33	0.34
	An gambiae	7	0.35	50	1.92	66	2.36	96	3.20	219	1.96
	An maculipalpis	30	1.5	0	0	0	0.00	54	1.80	84	0.83
	An nili	3	0.15		0	9	0.32	0	0.00	12	0.12
	An pharoensis	18	0.9	5	0.19	63	2.25	29	0.97	115	1.08
	An rufipes	20	1	17	0.65	26	0.93	0	0.00	63	0.65
	An squamosus	3	0.15	0	0	0	0.00	84	2.80	87	0.74
	An ziemanni	3	0.15	0	0	16	0.57	10	0.33	29	0.26
	An spp.	6	0.3	10	0.38	13	0.46	10	0.33	39	0.37
	TOTAL *Anopheles*	102	0.46	88	0.31	233	0.76	291	0.88	714	0.60
Culex	*Cx antennatus*	232	11.6	950	36.54	1450	51.79	1400	46.67	4032	36.65
	Cx bitaeniorhynchus	41	2.05	34	1.31	0	0.00	19	0.63	94	1.00
	Cx cinereus	0	0	0	0	0	0.00	60	2.00	60	0.50
	Cx decens	328	16.4	138	5.31	4	0.14	212	7.07	682	7.23
	Cx ethiopicus	33	1.65	5	0.19	0	0.00	18	0.60	56	0.61
	Cx neavei	107	5.35	26	1	24	0.86	623	20.77	780	6.99
	Cx perfuscus	113	5.65	141	5.42	44	1.57	217	7.23	515	4.97
	Cx poicilipes	6	0.3	17	0.65	451	16.11	108	3.60	582	5.17
	Cx quinquefasciatus	102	5.1	3331	128.12	38	1.36	1673	55.77	5144	47.58
	Cx univittatus	161	8.05	95	3.7	18	0.64	77	2.57	351	3.73
	Cx spp.	6	0.3	0	0	0	0.00	0	0.00	6	0.08
	TOTAL *Culex*	1129	5.13	4737	16.56	2029	6.59	4407	13.35	12 302	10.41
Mansonia	*Ma africana*	2359	117.95	1091	42.0	3416	122.00	2654	88.47	9520	92.59
	Ma uniformis	671	33.55	196	7.54	3483	124.39	420	14.00	4770	44.87
	Ma spp.	3	0.15		0	0	0.00		0.00	3	0.04
	TOTAL *Mansonia*	3033	50.55	1287	16.5	6899	82.13	3074	34.16	14 293	45.83
Number of mosquitoes per locality		4372	14.19	6291	8.53	9277	22.48	7911	12.23	27 851	14.36

No: Number of mosquitoes caught; M/T/N: Average number of mosquitoes collected per trap night (mosquitoes/trap/night).
